# Electron Tomography of Cryo-Immobilized Plant Tissue: A Novel Approach to Studying 3D Macromolecular Architecture of Mature Plant Cell Walls In Situ

**DOI:** 10.1371/journal.pone.0106928

**Published:** 2014-09-10

**Authors:** Purbasha Sarkar, Elena Bosneaga, Edgar G. Yap, Jyotirmoy Das, Wen-Ting Tsai, Angelo Cabal, Erica Neuhaus, Dolonchampa Maji, Shailabh Kumar, Michael Joo, Sergey Yakovlev, Roseann Csencsits, Zeyun Yu, Chandrajit Bajaj, Kenneth H. Downing, Manfred Auer

**Affiliations:** 1 Energy Biosciences Institute, University of California, Berkeley, California, United States of America; 2 Life Sciences Division, Lawrence Berkeley National Laboratory, Berkeley, California, United States of America; 3 Materials Sciences Division, Lawrence Berkeley National Laboratory, Berkeley, California, United States of America; 4 Department of Computer Science, University of Wisconsin, Milwaukee, Wisconsin, United States of America; 5 Department of Computer Sciences & The Institute of Computational Engineering and Sciences, University of Texas, Austin, Texas, United States of America; University of Aberdeen, United Kingdom

## Abstract

Cost-effective production of lignocellulosic biofuel requires efficient breakdown of cell walls present in plant biomass to retrieve the wall polysaccharides for fermentation. In-depth knowledge of plant cell wall composition is therefore essential for improving the fuel production process. The precise spatial three-dimensional (3D) organization of cellulose, hemicellulose, pectin and lignin within plant cell walls remains unclear to date since the microscopy techniques used so far have been limited to two-dimensional, topographic or low-resolution imaging, or required isolation or chemical extraction of the cell walls. In this paper we demonstrate that by cryo-immobilizing fresh tissue, then either cryo-sectioning or freeze-substituting and resin embedding, followed by cryo- or room temperature (RT) electron tomography, respectively, we can visualize previously unseen details of plant cell wall architecture in 3D, at macromolecular resolution (∼2 nm), and in near-native state. Qualitative and quantitative analyses showed that wall organization of cryo-immobilized samples were preserved remarkably better than conventionally prepared samples that suffer substantial extraction. Lignin-less primary cell walls were well preserved in both self-pressurized rapidly frozen (SPRF), cryo-sectioned samples as well as high-pressure frozen, freeze-substituted and resin embedded (HPF-FS-resin) samples. Lignin-rich secondary cell walls appeared featureless in HPF-FS-resin sections presumably due to poor stain penetration, but their macromolecular features could be visualized in unprecedented details in our cryo-sections. While cryo-tomography of vitreous tissue sections is currently proving to be instrumental in developing 3D models of lignin-rich secondary cell walls, here we confirm that the technically easier method of RT-tomography of HPF-FS-resin sections could be used immediately for routine study of low-lignin cell walls. As a proof of principle, we characterized the primary cell walls of a mutant (*cob-6*) and wild type Arabidopsis hypocotyl parenchyma cells by RT-tomography of HPF-FS-resin sections, and detected a small but significant difference in spatial organization of cellulose microfibrils in the mutant walls.

## Introduction

Sugars isolated from non-food plant biomass such as grasses or wood chips have the potential for large-scale biofuel production, but require efficient biomass deconstruction and fermentation for such fuel to be economically viable [1]–[4]. A variety of efforts are being undertaken to make these deconstruction processes efficient [1], [5]–[8] and the biomass less recalcitrant [2]–[4], [6], [9]–[10]. However, a more rational approach for both biomass deconstruction and feedstock development requires knowledge about the precise three-dimensional (3D) organization of plant cell walls, which are the storehouse of sugars in non-food biomass. Plant cell walls are composed of a relatively small number of carbohydrate- and phenolic-based building blocks, but the relative composition has changed throughout evolution, presumably accompanied by organizational changes in the wall architecture [11]–[12]. Plant cell walls are commonly distinguished as ‘primary cell wall’ or ‘secondary cell wall’, although a gradation of wall properties between these two extremes can be found in different cell types and various fundamentally different definitions have been proposed for the two terms to account for such diversity [13]–[15]. However, a primary cell wall may be defined as the extendable wall layer deposited in most growing cells, while secondary cell wall may be defined as the strong, non-extendable wall layers deposited between primary cell wall and plasma membrane in cell types such as, xylem tracheary element, fibers, and sclereids, after these cells stop growing [14]–[17]. Protoxylem is the only exception where secondary walls are deposited during cell growth, in arrangements that allow cell elongation. Secondary cell walls are not deposited in cell types such as cortical or vascular parenchyma cells, even at maturity [16]. Primary cell walls are composed of three groups of polysaccharides - cellulose, hemicelluloses and pectins, with few glycoproteins and enzymes also being present. Secondary cell walls are commonly composed of cellulose, hemicelluloses and large quantities of lignin as found in xylem tracheary element, fibers, and sclereids, although lignin-less secondary wall can be found in cell types such as phloem sieve elements, cotton fibers, and collenchyma cells. A thin cementing wall layer called the “middle lamella” separates the primary walls of two adjacent cells. Middle lamellae are mainly composed of pectins with small amount of hemicelluloses in the growing cells, and additional lignin deposited in the mature cell [16].

Cellulose is an unbranched polysaccharide of ß-1,4-glucose, with individual chains held together by hydrogen bonds to form long, unbranched structures called microfibrils. Microfibrils are reported to be ∼3–5 nm in diameter, consisting of multiple individual cellulose polysaccharide chains, with crystalline cellulose at the core surrounded by paracrystalline cellulose and hemicelluloses. The number of cellulose chains within a microfibril is debatable and has been reported to be 36 in older literature and 18–24 in recent years [18]–[25]. An ordered framework of microfibrils is thought to be the primary load-bearing structural feature of the wall, and the orientation of microfibrils plays a vital role in determining orientation of cell expansion [26]–[27]. Hemicelluloses are branched polysaccharides composed of neutral pentoses and hexoses that strengthen the cell wall by binding to cellulose via hydrogen bonds, and by binding with lignins in some cases [28]. Xyloglucan, a predominant hemicellulose in non-grass angiosperms, is widely believed to bind and surround the paracrystalline cellulose in the outer layer of microfibrils and to function as a tether between two neighboring microfibrils [22]–[23], [29]–[30]; however, the tether model has been challenged recently [31]. Pectins, like hemicelluloses, are branched polysaccharides, and contain high proportions of D-galacturonic acids. Their precise organization and respective functions are not fully understood [21], [29], [32]. Furthermore, glycoproteins and enzymes are also embedded in the cell walls, though their precise location is also unknown [21]–[22].

Most of our current knowledge about cell walls is based on indirect biochemical analyses of cell wall extracts, and the direct microscopy-based cell wall studies done to date give little insight into the 3D nature of the cell wall organization at macromolecular resolutions. Bulk orientation of cellulose microfibrils within the cell walls has been studied by polarized light, infrared microscopy [33]–[34], and confocal microscopy [35]–[36], but these methods have resolution of few hundred nanometers and cannot determine the architecture at individual microfibril level. Transmission electron microscopy (TEM) of surface replicas, prepared by rotary heavy metal shadowing of freeze-fractured, deep etched isolated cell walls samples, was the first method for visualization of cell wall architecture at high-resolution [37]–[40]. This method could however, focus only on a surface layer of the cell wall sample and was therefore restricted to studying only thin primary cell walls. Furthermore, this approach relies on biochemical isolation of cell wall material and involves substantial chemical treatment, and thus the cell walls may no longer be considered in close to their native state. Another remarkable TEM study done in the 1970s focused on the architecture of unisolated cell walls using two complementary sample preparation methods [41]. Parenchyma as well as collenchyma cell walls were mildly extracted and stained with polysaccharide specific stain: periodic acid-thiocarbohydrazide-silver proteinate (PATAG) to understand the cytochemistry; and the fine ultrastructure of comparable walls were studied by chemically fixing, liquid nitrogen freezing, cryo-sectioning and negatively staining of sections with sodium silicotungstate. Although the findings of this study is relevant even to current date, the presence of fixatives and stains in the cryo-sectioned samples leave a doubt that these chemicals might have altered the wall organization at macromolecular level. Three-dimensional texture of the walls was also evaluated in this study by tilting the stage from −45° to +45°, which can be considered as precursor of electron tomography. However, this method does not provide true 3D information of inside the cell wall sections. In recent years, field emission-scanning electron microscopy (FE-SEM) and atomic force microscopy (AFM) have been used to study the organization of cell wall components, especially the cellulose microfibrils at high resolutions [23], [42]–[46]. These studies have yielded valuable topographical surface information, but neither of these techniques allows access to 3D architectural information.

Electron tomography is the only method currently available that can provide true 3D visual insight into plant cell wall ultrastructure at macromolecular resolution (∼2 nm) without isolating the cell walls. Electron tomography relies on the collection of single- or dual-axis tilt series, each containing ∼70–280 TEM images recorded at different tilt angles of the specimen in the electron microscope. After alignment of individual images with the help of fiducial markers, and 3D reconstruction using weighted back-projection techniques [47]–[48], 3D volumes (tomograms) showing exquisite detail in 3D are obtained. Such tomograms need to be visually inspected and features of interest must be segmented out before geometrical parameters such as distances, volumes, angles, and lengths can be determined and a realistic 3D model can be built. Electron tomography has been used in recent years in plant biology to study the 3D ultrastructure of the secondary cell wall of *Pinus* wood tissue [49]–[50] and to characterize plant cell wall deconstruction during thermo-chemical pretreatment of corn stover biomass [51]–[52]. However, for these studies, the plant samples were chemically fixed and dehydrated in organic solvents at room temperature, and included additional harsh chemical treatment to remove lignin, before embedding the samples in resin. Such sample preparation protocols can lead to aggregation and extraction artifacts as well as uneven or preferential staining that can profoundly alter the perception of the organization [53].

In this paper, we present two new approaches of studying the macromolecular 3D ultrastructure of plant cell wall that include electron tomography of cryo-immobilized fresh tissue, and avoid the conventionally used harsh chemical treatments. We show that faithfully preserved cell walls can be obtained by self-pressurized rapid freezing (SPRF) of fresh tissue followed by cryo-sectioning, as well as by high-pressure freezing (HPF), freeze-substitution (FS) and resin embedding. With cryo-electron tomography of the unstained cryo-sections of intact unextracted Arabidopsis tissue, we were able to visualize never seen before details of macromolecular 3D architecture of both lignin-less primary cell walls and the lignin-rich secondary cell walls in situ in their near-native state. We also show that high-quality 3D data of lignin-less primary cell walls can be obtained by using the relatively easier method of room temperature (RT) electron tomography of HPF-FS-resin embedded, stained sections. Even though cryo-immobilization approaches have been used to address various biological questions, electron tomographic study of plant cell wall architecture using either of the two cryo-immobilization approaches has never been reported to our knowledge. Our cryo-tomography approach will be the first reported imaging method to visualize the organization of polysaccharides at macromolecular (∼2 nm) resolution in unextracted lignin-rich secondary cell walls. Using a semi-automated threshold-based segmentation method we further analyzed relatively larger cell wall volumes qualitatively as well as quantitatively, which has not been done for any previous electron tomography study of plant cell walls. As an example of potential routine application of electron tomography of cryo-immobilized plant cell walls, we characterized the subtle architectural differences in the primary cell walls of mutant (*cob-6*) Arabidopsis hypocotyl parenchyma cells, compared to those of wild type (Col 0), as *cob* mutants have been reported to cause disorganization of cellulose microfibril orientation and reduction of crystalline cellulose in the cell walls of roots [54]–[56].

## Materials and Methods

### Plant material

For comparison of sample preparation methods, wild type *Arabidopsis thaliana* (Arabidopsis) seeds from the Colombia ecotype (Col 0) were sterilized in 30% bleach, 0.02% Triton and vernalized at 4°C in water for 48 hours. They were germinated on 0.7% agar plates containing 0.5x Murashige and Skoog medium for 10 d at 21°C under continuous light in a growth chamber. The seedlings were then transferred to pots containing soil mixture and placed in a growth chamber programmed for a 16 h light/8 h dark cycle at 21°C. Stem tissue from 3–4 weeks old plants that had newly growing inflorescence stems (2–3 cm long) were used for the three different sample preparation methods described below. For comparative analysis, cell wall areas from all sample types were randomly selected for electron tomography, from cells within xylem tissue that appeared to be xylem tracheary elements.

### Self-pressurized rapid freezing (SPRF), vitreous sectioning and cryo-electron tomography

**Figure 1:**
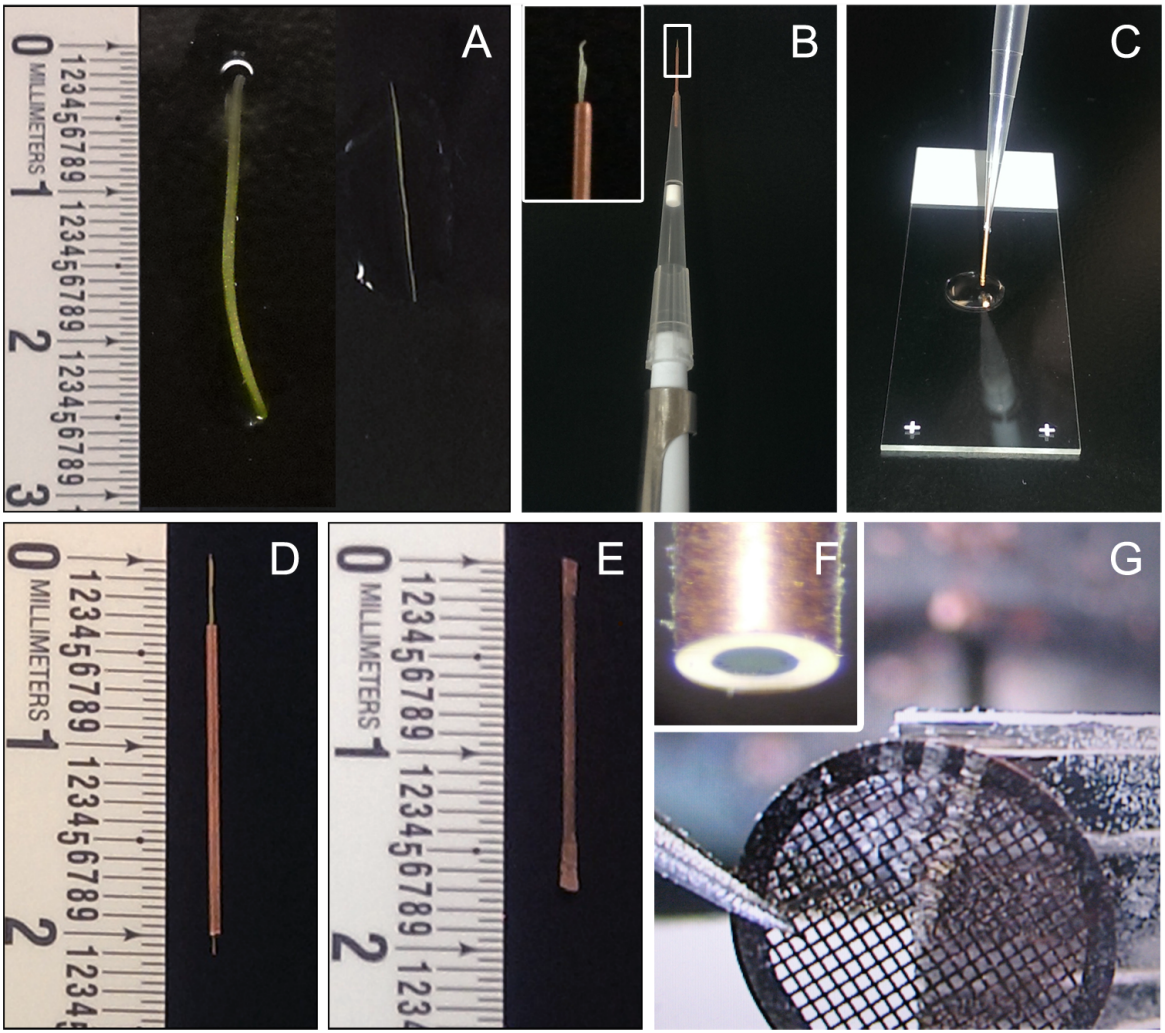
Steps of self-pressurized rapid freezing (SPRF) and cryosectioning of plant tissue. A. Intact stem segment (left) and a dissected longitudinal ‘hair-thin’ segment of stem tissue (right). B. Tissue inserted on one end of a copper capillary tube, fitted to a pipette on the other end (inset showing close-up). C. Insertion of tissue into the capillary tube by pipetting in cryo-protectant solution. D. Stem segments inserted completely into a capillary tube. E. Capillary tube with sample and cryo-protectant sealed from both end. F. Trimmed capillary tube with frozen sample inside. G. A ribbon of cryo-sections being collected on TEM grids.

Young inflorescence stems (2–3 cm long) were rapidly hand sectioned with a sharp razor blade into longitudinal ‘hair-thin’ segments (Fig. 1A). Each tissue segment was then inserted into individual 15 mm long clean, deoxidized copper capillary tubes (inner and outer diameter of 300 µm and 600 µm respectively), in a manner very similar to threading a needle. The capillary tube was then fitted to a pipette with the sample side facing outward (Fig. 1B) and a cryoprotectant solution (20% dextran) was sucked into the capillary tube (Fig. 1C) until droplets of dextran came out from the other end of the tube. The dextran solution fills the air pockets around the stem and reduces freezing damage, and at the same time helps in pulling the sample all the way up into the capillary tube. Due to the small diameter of the tubes, the sample insertion process was difficult, with most stems being stuck within the tubes and not coming out of the other end. All such samples were discarded. Only those stem segments that smoothly came out of the other end (Fig. 1D) were used for further steps to ensure minimum handling damage. Unlike resin sections, cryo sections cannot be quickly checked for presence of samples at the time of sectioning. To minimize chances of getting blank sections, we used only those capillary tubes that had one long stem tissue spanning the entire length of the tube. More than one short stem segment can be inserted back to back instead, if ease of tissue insertion is preferred over ease of finding the sample within the sections. The sample-containing capillary tubes were then quickly sealed at both ends, one end at a time, with a pair of pliers to build pressure inside the tubes (Fig. 1E). The sealed tubes were immediately plunged sideways in liquid ethane for self-pressurized rapid freezing [57]–[60] of the stem tissue. It was crucial to do all these steps very quickly in order to minimize tissue damage. The sealed tubes were then stored in liquid nitrogen until cryo-sectioning.

Prior to cryo-sectioning, both sealed ends of each tube were trimmed off under liquid nitrogen using a cryo-trimmer diamond knife (Diatome, Hatfield, PA, USA), leaving the central uncompressed part of the tubes with frozen tissue (Fig. 1F). Transverse sections of a nominal thickness of 70 nm were obtained by cryo-sectioning the frozen samples at −160°C using a Leica EMUC6 ultramicrotome fitted with a Leica EM FC7 cryo chamber attachment (Leica Microsystems Inc.), and a 25° cryo-diamond knife for least possible compression (Diatome, Hatfield, PA, USA). Vitreous cryo-sections were picked on carbon-coated lacey/formvar copper mesh grids (Ted Pella, Redding, CA, USA; Fig. 1G and Video S1) and attached to the grids with a Leica EM CRION ionizer [61]. The cryo-sections were screened using very low dose techniques for selecting areas of interest for tomography at low magnifications to avoid beam damage to the delicate cryo-sections. The very low contrast of cryo-sections makes it challenging to do proper anatomical distinction during rapid screening. Since our objective for this study was to only standardize the method, we randomly chose areas with thick secondary cell walls that are presumably xylem tracheary elements. Single-axis cryo tilt series were then collected from −60° to +60° with 2° increments at higher magnification under low dose conditions. Tilt series were recorded by using a Tecnai F20 FEG TEM (FEI Company, Hilsboro, OR, USA) equipped with a cold stage (Gatan, Pleasanton, CA, USA), operated at 120 kV. Images were recorded with a 4K×4K Gatan Ultrascan 4000 CCD camera (Gatan, Pleasanton, CA, USA) and the automatic data acquisition system Leginon [62] at a final magnification of 25,000x corresponding to a detector pixel size of 0.43 nm at the specimen level, and a defocus set to −6 micron. Alternatively, tilt series were recorded using a JEOL JEM–3100 FFC FEG TEM (JEOL Ltd, Akishima, Tokyo, Japan) equipped with an in-column Omega energy filter and a cryo- transfer stage, operated at 300 kV. Zero-loss images were recorded with a 30 eV energy selecting slit, using a Gatan 795 2K×2K CCD camera (Gatan, Pleasanton, CA, USA) and SerialEM software [63], at a final magnification of 25,000x corresponding to a pixel size of 1.1 nm at the specimen level and a defocus set to −12 micron.

### High-pressure freezing (HPF), freeze substitution (FS)

Transverse free-hand sections (<200 µm thick) taken from approximately the middle part of the 2–3 cm long young inflorescence stem were placed in 200 µm deep freezer hats (Ted Pella, Redding, CA, USA) with 1-hexadecene as cryoprotectant, high pressure frozen in a Leica EMPACT2 high-pressure freezer (Leica Microsystems Inc.), and then transferred to liquid nitrogen. The samples were then processed following a modified version of previously published freeze-substitution, resin-embedding protocols for plant samples [64]–[67]. The frozen samples were freeze substituted in 2% OsO_4_ and 0.5 mg/ml ruthenium red in anhydrous acetone at −90°C for 5 d, followed by slow warming to room temperature (RT) over a period of 2 d, in the Leica AFS2 (Leica Microsystems Inc.). After rinsing in several acetone washes, the samples were removed from the holders, and infiltrated with increasing concentrations of Epon-Araldite resin (Ted Pella, Redding, CA, USA) in acetone according to the following schedule: 4 h in 5% resin, 4 h in 10% resin, 12 h in 25% resin, and 24 h in 50%, 75%, and 100% resin, respectively. Polymerization was performed at 60°C for 2–3 days.

### Conventional chemical preparation

Stem segments (∼2 mm long) from approximately the middle of the 2–3 cm long young inflorescence stem were fixed with 4% paraformaldehyde, 2% glutaraldehyde in 0.03 M phosphate buffer (pH 7.4) containing 0.5 mg/ml ruthenium red overnight at 4°C. After washing with the same buffer, secondary fixation was done with 0.1% osmium tetroxide and 0.5 mg/ml ruthenium red for 1 h at RT. The samples were then dehydrated and infiltrated at RT using a Leica EM AMW automatic microwave tissue processor (Leica Microsystems Inc.). Dehydration was done in ethanol series (25%, 50%, 75%, 95%, 100%, twice in each solution for 45s each), followed by dehydration in 100% acetone (twice for 45s each). The samples were then infiltrated in Epon-Araldite resin-acetone series (5%, 10%, 25%, 50%, 75%, thrice in each solution for 3 min each), infiltrated in 100% resin on a rotor at RT overnight, followed by 3 h infiltration in 100% resin with accelerator, and finally polymerized at 60°C for 2–3 days.

### Ultrathin sectioning and room temperature electron tomography of resin embedded samples

Thick transverse sections (∼150 nm) were cut from both chemically prepared and HPF-FS samples using the Leica UC6 ultramicrotome (Leica Microsystems Inc.). Sections were picked on formvar-coated copper slot grids (Ted Pella, Redding, CA, USA). All sections were exposed to 5 nm gold fiducials for 4 mins each on both sides followed by several washes in distilled water. The sections were then post stained with 2% uranyl acetate in methanol for 5 mins, followed by Reynold’s lead citrate solution for 2 mins. Cell walls of xylem tracheary elements comparable to those selected for cryo-tomography were selected for RT tomography based on cell shape, size, location and wall thickness. Dual axis tilt series were collected from +65° to −65° with 1° increment on a Tecnai F12 FEG TEM (FEI Company, Hilsboro, OR, USA) operated at 120 kV accelerating voltage and equipped with Fischione dual-axis tomography holder (Fischione Instruments, Pittsburg, PA, USA). Images were collected with a 2K×2K Gatan Ultrascan 1000 CCD camera (Gatan, Pleasanton, CA, USA) and SerialEM software [63], [68], at a final magnification of 11,000x or 13,000x corresponding to a pixel size of 0.79 nm or 0.92 nm, respectively at the specimen level, and a defocus set to −1 micron.

### Image alignment, 3D reconstruction and filtering

Images were aligned and reconstructed with IMOD (The Boulder Laboratory for 3D Electron Microscopy of Cells, University of Colorado Boulder, CO, http://bio3d.colorado.edu/imod/) using the back-projection method [68]–[69]. All fiducial-less cryo-tomograms were aligned by the ‘patch-tracking’ method while the resin sections with gold fiducial markers were aligned by the ‘fiducial-tracking’ method, both available in the IMOD package. The cryo-tomograms collected with a 4K×4K camera were binned by 2 to obtain a pixel size of 0.87 nm, for easy comparison with all other tomograms. All cryo-tomograms were subjected to image filtering using the nonlinear anisotropic diffusion (NAD) filter [70] within the IMOD package, to reduce noise and improve contrast for ease in segmentation.

### Selection of segmentation approach

In order to minimize/eliminate any bias that might be introduced from using any one specific feature extraction approach, we first tested three different protocols, namely, manual segmentation, semi-automated threshold segmentation, and an automated custom-built algorithm-based segmentation approach, to extract the cell wall features from a small 3D volume of a tomogram. The manual approach involved precise but labor-intensive tracing of wall features by visual inspection using the commercially available imaging program Amira (Visualization Sciences Group, Burlington, MA, USA). For the semi-automated method, the ‘threshold segmentation’ tool available in Amira was used, where a threshold value has to be selected by visual inspection so that only the cell wall features denser than the general background are segmented out. For both of the above-mentioned approaches, triangular mesh surfaces were generated from the segmented images with the ‘unconstrained smoothing’ option in Amira for better visualization of segmented wall features in 3D. For the custom-built automated approach, each voxel in the NAD filtered volume was associated with a pre-computed local structure tensor [71]–[73] that defines the local feature orientation. A Gaussian-like filter was anisotropically applied to the neighborhood of each voxel in such a way that smoothing was favored in the direction of the local feature orientation given as the eigenvector corresponding to the smallest eigenvalue of the tensor. The filtered volumes were then segmented by topological isosurface selection via the contour spectrum [74] and skeletons were extracted with a topology-preserving thinning algorithm [75] on the segmented volumes. We found no significant difference in the segmented volumes for these very different approaches (Fig. S1A–G), but the semi-automated threshold approach in Amira was the easiest to use. We further tested reliability of the threshold approach by segmenting the same volume with small variations in threshold values, the same volume by 2 different individuals independently, and three different tomograms of three different cell wall samples prepared by the same sample preparation method. After finding consistently reliable threshold ranges for unstained cryo and stained resin samples (Fig. S2A–D, details in Results section), we used the semi-automated threshold segmentation approach for rest of our analyses.

### Segmentation and quantitative image analysis of 3D volumes from primary cell walls

For comparative segmentation and quantitative image analysis, we selected nine 3D volumes (500 pixels×500 pixels×Z, where Z = 50, 75, or 100, depending on the number of image slices with clearly distinguishable biological material in each tomogram) from cell wall areas visible in three different tomograms (three volumes from each tomogram) for each of the three sample preparation methods. Cell wall features were then segmented and mesh surfaces were generated for all tomogram volumes by the threshold segmentation approach described above. For quantitative geometric analysis of the cell wall components, we randomly picked two planes within each 3D volume and measured the cross-sectional diameter of randomly picked long filamentous structures, their separation (center-to-center distance) as well as the shortest gap between the filaments (edge-to-edge distance), and the cross-sectional length of short bridge-like cross-links joining the long and typically parallel-running filaments in only the primary cell wall area of each tomogram volume. Secondary cell walls were not included in quantitative analysis, as they appeared featureless in resin-embedded samples.

### Chemical treatment - for identification of cell wall features

Wild type Arabidopsis (Col 0) seeds were sterilized and germinated on media, seedlings were transferred and grown in soil for ∼3 weeks, and young inflorescence stem segments were fixed, dehydrated, and resin embedded by conventional chemical method, as described above. An additional chemical treatment was done in between the two fixation steps, where stem segments were treated with 0.5% ammonium oxalate at 60°C for 48 hours followed by 4% NaOH at RT for 96 h to remove pectin, hemicelluloses and any non-cellulosic polysaccharides from the cell walls. The extracted samples were sectioned; and cell walls areas were imaged, reconstructed, segmented and analyzed as done for all resin embedded samples above, and compared with non-extracted controls.

### Mutant characterization – a proof of concept for possible routine analyses

Wild type Arabidopsis (Col 0) and mutant (*cob-6*) seeds were sterilized and germinated on media as described above. Transverse free-hand sections (<200 µm thick) from the hypocotyls of ∼1 week old plants were prepared by the HPF-FS method described above. The samples were sectioned, imaged, reconstructed, segmented and analyzed as done for the HPF-FS samples above, except primary cell walls of cortical parenchyma cells were selected from both WT and mutant samples for this comparative analysis. For each sample, three cell wall 3D volumes (500 pixels×500 pixels×Z, where Z = 50, 75, or 100, depending on the number of image slices with clearly distinguishable biological material in each tomogram) were used for the quantitative analysis.

## Results

We present the results of a comparative study of Arabidopsis cell wall 3D ultrastructure obtained by three different methods: (1) Cryo-tomography of unstained vitrified cryo-sections cut from self-pressurized rapidly frozen (SPRF) samples, (2) Room temperature (RT) tomography of stained sections of high pressure frozen (HPF), freeze substituted (FS), resin-embedded samples, and (3) RT tomography of stained sections of microwave-assisted chemically fixed, dehydrated and resin-embedded samples (equivalent to a conventional bench-top protocol).

### Comparison of sample preservation quality by 2D transmission electron microscopy (TEM)

**Figure 2:**
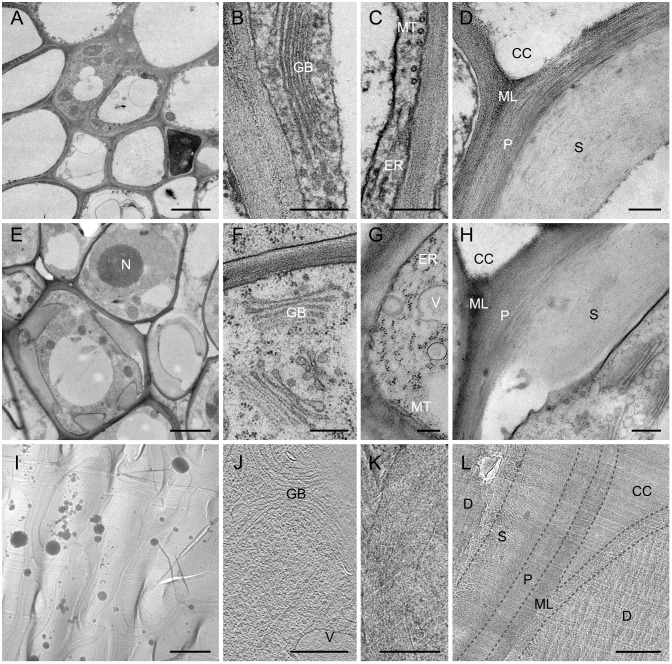
Comparison of sample preparation quality of Arabidopsis stem tissues by transmission electron microscopy. A–D. Chemically prepared samples (conventional method). E–H. High pressure frozen, freeze substituted samples. I–L. Vitreous sections of self-pressurized, rapid frozen samples. A, E, I. Cells and organelles at below 1000x. Bars = 2 µm. B–C, F–G, J–K. Cell organelles at over 10,000x. Bars = 250 nm. D, H, L. Cell wall layers at over 10,000x (Bars = 250 nm). Dotted lines in L. shows the limits of cell wall layers in the low contrast vitreous section. CC = Cell corner, D = Damaged areas, ER = Endoplasmic reticulum, GB = Golgi Bodies, MT = Microtubules, ML = Middle lamella, N = Nucleus, P = Primary cell wall, S = Secondary cell wall, V = Vesicles.

Using a 3-day long conventional chemical preparation that included microwave-assisted chemical fixation, dehydration and resin embedding, we obtained fair preservation of Arabidopsis stem tissue (Fig. 2A–D). Cell membranes and most organelles such as nuclei, chloroplasts, mitochondria, Golgi bodies, vesicles, ribosomes, endoplasmic reticulum, and microtubules were preserved (Fig. 2A–C), although the quality of preservation was inferior to the samples prepared by the two cryo-immobilization methods as evidenced by wavy membranes (Fig. 2B, C). We found overall morphology of the cell walls in these samples to be intact without signs of breakage or deformation. The different layers of the cell wall showed different levels of staining, with the middle lamella being the darkest and the secondary cell wall being the lightest (Fig. 2D). When imaged at magnifications over 10,000x, patterns of alternating dark and light filamentous structures were clearly visible in the primary cell walls (Fig. 2D). The secondary walls, however, appeared to be homogeneously light without any detectable texture (Fig. 2D), suggesting that the contrast-generating heavy atom staining solutions did not reach or react with the secondary cell wall material.

High-pressure freezing, freeze substitution (HPF-FS) was carried out with a modified version of a widely reported ∼2 week long general protocol for plant samples [64]–[67]; this provided a much higher quality of preservation of Arabidopsis stem tissue (Fig. 2E–H) compared to the chemical preparation protocol (Fig. 2A–D). Cell membranes and organelles including nuclei, chloroplast, mitochondria, Golgi bodies, ribosomes, endoplasmic reticulum, vesicles, and microtubules appeared to be well preserved (Fig. 2E–G). As was the case for the chemically prepared samples, the cell walls of the HPF-FS samples did not show damage and the different layers of the wall appeared equally differentially stained (Fig. 2H). Upon visual inspection, the primary cell walls of HPF-FS samples appeared to be much more densely packed when imaged at magnifications over 10,000x (Fig. 2H), compared to those in the microwave-assisted chemically prepared samples (Fig. 2D), with a dense pattern of lightly and darkly stained filamentous structures. Secondary walls of HPF-FS samples, on the other hand, appeared light and rather featureless (Fig. 2H), similar to the chemically prepared samples (Fig. 2D).

Upon adaptation of the novel approach of using the self-pressurized rapid freezing (SPRF) method [57]–[60] to freeze plant samples, vitreous sectioning of the frozen samples, and imaging by cryo-TEM [61], [76]–[77], we succeeded in obtaining several good areas of vitreous Arabidopsis stem tissue sections (Fig. 2I–L). Freezing in capillary tubes provided better sample height during cryo-sectioning compared to using freezer hats that are commonly used for HPF, which made it relatively easier to obtain vitreous sections from the samples. We found that simple insertion of the plant stem segments into unsealed copper capillary tubes and freezing by a standard high-pressure freezer trapped air bubbles within the frozen sample, often resulting in poor preservation of the samples. We overcame this problem by injecting 20% dextran solution into the capillary tubes and manually applying pressure to seal the capillary tube from both sides; this seemed to remove most of the air bubbles trapped around the stem within the capillary tubes in 1 out of every 3 tubes prepared. As our high-pressure freezer was not equipped to freeze sealed capillary tubes, we used liquid ethane plunge freezing, as is typically performed on ultrathin whole mount samples [78]–[79]. The tubes that were mostly air-bubble free, froze well, although freezing was not always uniform throughout the entire tissue segment, possibly due to factors such as uneven thickness of sample, uneven distribution of dextran, and air trapped within the plant tissue. Only well-frozen samples that produced continuous long ribbons of intact sections were considered “good grids” and used for imaging. Although we frequently encountered a large variety of artifacts, including a waviness of the sections causing compression of the cell in the direction of cutting, occasional breaks, knife marks and contamination with electron-dense ice particles (Fig. 2I) even within the good grids, we found small (∼100–200 µm) patches of flat and uncompressed areas all over each section in such grids. The cell walls in such uncompressed areas appeared to be undamaged (Fig. 2L) and only such apparently good cell wall areas that also showed details of primary and secondary cell walls in the projection view were selected for 3D imaging. We collected 23 tilt-series from 5 good grids, each of which had 2–3 ribbons of ∼20 apparently flat, undamaged sections. Success rate of getting good alignment and reconstruction of these fiducial-less tilt-series varied due to section waviness or breaks within the region imaged. The 3 data sets that aligned and reconstructed well (error values within acceptable range recommended in IMOD package) and showed unprecedented detail of both primary and secondary cell walls after filtering and segmentation were used for qualitative analysis. Our results have established that cryo-tomography of unisolated, unextracted plant tissue sections can visualize plant cell walls in their near-native frozen-hydrated state. While the cell organelles could not be distinguished at the low magnifications used to survey the grids (Fig. 2I), some organelles such as Golgi bodies and vesicles became visible in some parenchyma cells when imaged at magnifications above 10,000x (Fig. 2J). Other organelles such as microtubules, ribosomes, and endoplasmic reticulum could not be detected with certainty in the sections surveyed due to the low contrast typical to the cryo-images (Fig. 2K). At high magnification, cell wall components appeared densely packed, albeit with low contrast due to the absence of any heavy metal electron-dense staining. We could clearly distinguish the different layers of the cell wall – middle lamella, primary cell wall, and secondary cell wall, with the middle lamella appearing to be much denser than other layers of the wall. Furthermore, we detected well-organized filamentous structures in both primary cell walls and secondary cell walls (Fig. 2L).

### Electron tomography and threshold segmentation of plant cell walls

**Figure 3:**
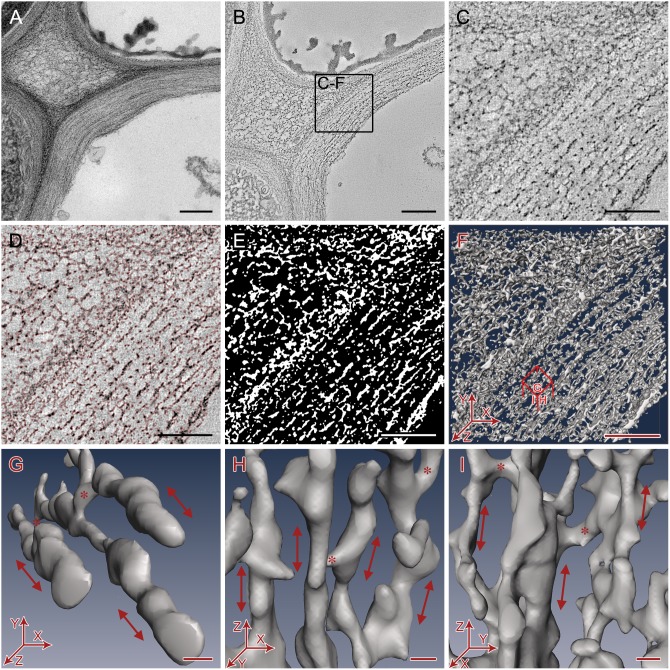
The electron tomography data collection and segmentation process used on Arabidopsis primary cell walls randomly chosen from chemically fixed samples. A. 2D projection image of cell wall. B. Slice of reconstructed tomogram. Bars = 250 nm. C. Sub-area of tomogram. D. Electron dense cell wall components selected by thresholding (selected areas outlined in red). E. Segmentation map of thresholded cell wall components (white). F. Mesh surface rendering of threshold segmentation map. Bars = 100nm. G-I. Small representative 3D volumes of the segmented cell wall showing orientation of filamentous cellulose microfibrils (arrow) and hemicellulose cross-connections (*). G- Top view showing microfibrils running approximately along the axis of cell elongation (Z-axis). H- Side view showing a single layer of microfibrils. I - Side view showing neighboring layers of microfibrils. Bars = 5 nm.

For each sample type, we selected comparable cell wall areas based on cell shape, size, location and wall thickness, and followed identical steps of electron tomography and image analysis. Figure 3 illustrates these steps in a randomly chosen primary cell wall area of a conventionally prepared sample, as an example. Once we identified any promising area with cell walls suitable for tomographic 3D imaging (Fig. 3A), we collected single- or dual-axis tilt series images and reconstructed them into 3D volumes (Fig. 3B, Video S2). Inspection of such 3D volumes, ∼1 nm slice at the time, at full resolution revealed that electron-dense cell wall components were arranged in layers that are nearly parallel to each other (Fig. 3C). Using the threshold segmentation tool in Amira, with a threshold chosen to include most of the electron-dense features for each slice above background noise (Fig. 3D), a segmented map was obtained (Fig. 3E) that could be rendered as a mesh surface for 3D visualization (Fig. 3F, Video S3).

Closer inspection of 3D volume (Fig. 3F) and smaller sub-volumes from different orientations (Fig. 3G–I) revealed a layered arrangement of filamentous primary cell wall components, oriented nearly parallel to the plasma membrane running along the direction of the cell elongation. The long filamentous structures observed in our segmented tomograms match the previously published description of cellulose microfibrils as long, unbranched filaments of ∼3–5 nm diameter [21], [23], although we noticed that these filamentous structures do not appear to be as smooth as seen by previous microscopy studies. The filamentous structures detected by our method likely include a crystalline cellulose core surrounded with para-crystalline cellulose (termed elementary fibril by Ding and Himmel, [23]) that possibly has some hemicelluloses and/or pectins attached as well. Following the convention started by Ding and Himmel [23], we will refer to the filamentous structures detected in our tomograms as ‘microfibrils’ in the remaining text. It must also be noted that the microfibrils in our images appear relatively short compared to previously published length of several microns for cellulose microfibrils because of section geometry, with most microfibrils spanning only the 100–150 nm thickness of each microtome section. In all samples, microfibrils were widely separated from other microfibrils within the same layer (Fig. 2G–H) as well as those in the neighboring layers (Fig. 3I). Bridge-like densities were frequently found between the microfibrils within the same layer as well as between neighboring layers (Fig. 3G–I), which were absent in cell walls of samples extracted with ammonium oxalate and sodium hydroxide that remove hemicellulose and pectin (Fig. S3A–C). We refer to such densities as ‘cross-links’ in the remaining text as they somewhat match the description of hemicellulose cross-links shown in the well-accepted cell wall models [21], [38]. However, these densities could also be pectins as suggested by a recent NMR-based cell wall model [80]. The exact chemical identity, the nature of their chemical interactions and the mechanical roles of these densities cannot be determined from our current data, but can be studied in future using cell wall mutants. The ‘empty space’ between the electron-dense microfibrils and cross-links could be filled with something that does not get stained (in case of stained sections) or have densities closer to ice (in case of unstained cryo-sections), which could include gases or water in primary walls (that was replaced by resin or amorphous ice in the sections) or lignin in secondary walls.

### Qualitative comparison of cell wall preservation quality in electron tomograms

**Figure 4:**
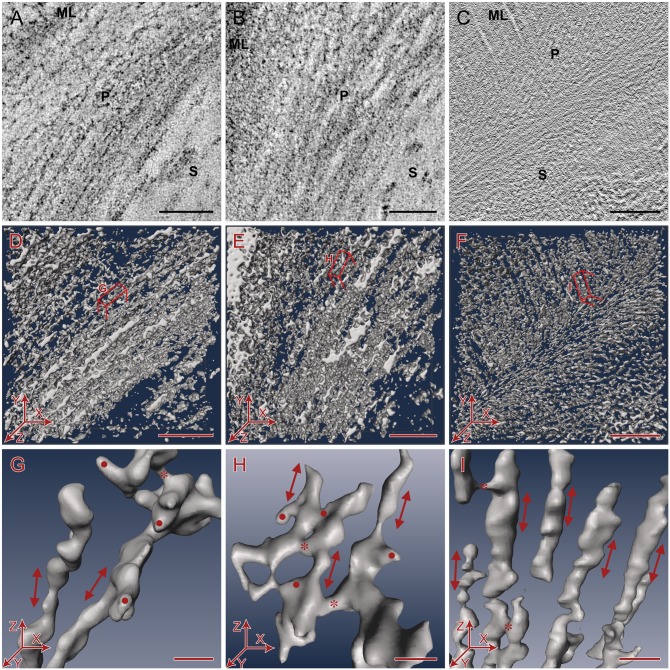
Qualitative comparison of cell wall preservation quality in electron tomograms. A–C. 2D slices taken from the 3D electron tomograms showing overview of cell wall ultrastructure in transverse sections. Bars = 100 nm. D–F. Overview of segmented cell wall volumes. Bars = 100 nm. G–I. Close-up of segmented primary cell wall volumes showing arrangement of cellulose microfibrils (red arrow), cross-links (*), and additional density artifacts (•) from a side view (XZ plane) of the tomograms. Z-axis is approximately the axis of cell elongation. Bars = 10 nm. A, D, G. Chemically prepared samples (conventional method). B, E, H. High pressure frozen, freeze substituted samples. C, F, I. Vitreous sections of self-pressurized, rapid frozen samples. ML = Middle lamella, P = Primary cell wall. S = Secondary cell wall.

Inspection of individual slices of the raw tomogram (Fig. 4A–C) as well as the threshold segmented density map (Fig. 4D–F) revealed less material in the primary cell wall in samples prepared by conventional chemical protocol (Fig. 4A, D) compared to the HPF-FS-prepared samples (Fig. 4B, E), suggesting that some cell wall components get extracted during the conventional protocol. While in comparison to primary cell walls the pectin-rich middle lamellae appeared denser in both chemically prepared and HPF-FS samples, it was much denser in HPF-FS samples (Fig. 4D–E). At a threshold where the individual structures in the primary cell walls could be segmented well in the HPF-FS samples, the fine features of the middle lamellae could no longer be resolved (Fig. 4E), indicating retention of large amount of pectins and hemicelluloses. Regarding the fine structures of secondary cell walls of both chemically prepared and HPF-FS samples, 2D projection and electron tomography studies did not reveal any fine structural details. However, the filamentous structures in both primary and secondary cell walls were readily visible in the unstained vitreous sections imaged by cryo-electron tomography (Fig. 4C), despite the overall lower contrast of unstained samples. In segmented cryo-tomograms, we were able to detect a highly organized 3D architecture in all layers of the cell wall including the pectin rich-middle lamellae, the primary cell walls, as well as the lignin-rich secondary cell walls (Fig. 4F). To maintain consistency, we restricted the quantitative analysis to the primary cell walls for all sample types.

Apart from differences of the various sample preparation approaches in the overall density of cell wall macromolecules we also noticed that in various parts of the conventionally processed samples, the microfibrils ran in somewhat random directions and occasionally crossed or connected with neighboring microfibrils within the same layer or the neighboring layers (Fig. 4G). In contrast, we found the overall orientation of the microfibrils and layers within the cell wall to be more consistently parallel in the HPF-FS preserved (Fig. 4H) and thus in agreement with cryo-preserved, unstained cell walls (Fig. 4I), suggesting higher order and thus better preservation of the sample in both cryo-immobilization methods compared to conventionally processed samples. The microfibrils in the chemically prepared and HPF-FS samples frequently had additional densities with variable shapes attached to them such as non-filamentous structures, blobs of dense material attached to microfibrils without any obvious pattern, or wide flattened stretch of densities between microfibrils (Fig. 4G, H) that were not present in the cryo-preserved samples (Fig. 4I), which most likely are artifacts from either aggregation of macromolecules or the use of electron-dense stains. The areas with such uncharacteristic additional densities were carefully excluded from the quantitative analyses.

### Quantitative geometric analysis of cell wall preservation quality in electron tomograms

Qualitative as well as quantitative analysis of slices of the segmented tomograms showed that the diameter of microfibrils varied significantly along individual microfibrils for all sample preparation schemes (Fig. 4D–F, 5A). We set out to quantify such differences although we realize that (1) there are limitations imposed by the respective sample preparation approaches, e.g. the possibility of preferential staining, (2) the best resolution achievable by electron tomography imaging of ∼100–150 nm thin sections is limited to no better than ∼2 nm, and (3) it is difficult to establish an objective density threshold, particularly when comparing different data sets. However, for any given data set, all data can be thresholded at the same value allowing a comparison of different microfibrils and cross-links within each data set. We typically applied threshold at a density value where we could visually distinguish the microfibrils and cross-links from background, the microfibrils generally appeared to be long and continuous, and the cross-links clearly bridging two microfibrils. The Segmentation Editor window in Amira software allows simultaneous visual inspection of threshold label image and the original density image, which helps the user to avoid selecting a significantly lower threshold that would include a substantial fraction of noise, or a much higher threshold that would result in disconnected isolated density. We found that selecting a threshold value around ‘0’ in the Amira interface consistently segmented the visible density accurately. A small variation (0±25) yielded essentially the same measurements (Fig. S2A). A larger variation (0±75) resulted in an overall shift of 1–1.5 nm that corresponds to a difference of 1–2 pixels (Fig. S2B), but such differences were consistent between different data sets. Even when 2 different individuals independently chose ‘ideal threshold values’ and measured independently chosen microfibrils, the overall results were remarkably similar with maximum difference of ∼1 nm (Fig. S2C). Such small differences in results were not accounted as significant differences in any of our comparative studies. Even though density maps cannot be completely objectively thresholded for different data sets, a comparison of measurements of microfibril diameter from the same sample preparation protocols but in different data sets yielded remarkably similar numbers (Fig. S2D), suggesting that a comparison between different sample preparation protocols is legitimate, and the same threshold approach can be used to measure cross-link length and edge-to-edge distances between microfibrils as well. Moreover, the above-mentioned limitations do not affect the center-to-center distances of the microfibrils and hence, any significant difference in filament spacing between data sets must therefore represent a real difference.

**Figure 5:**
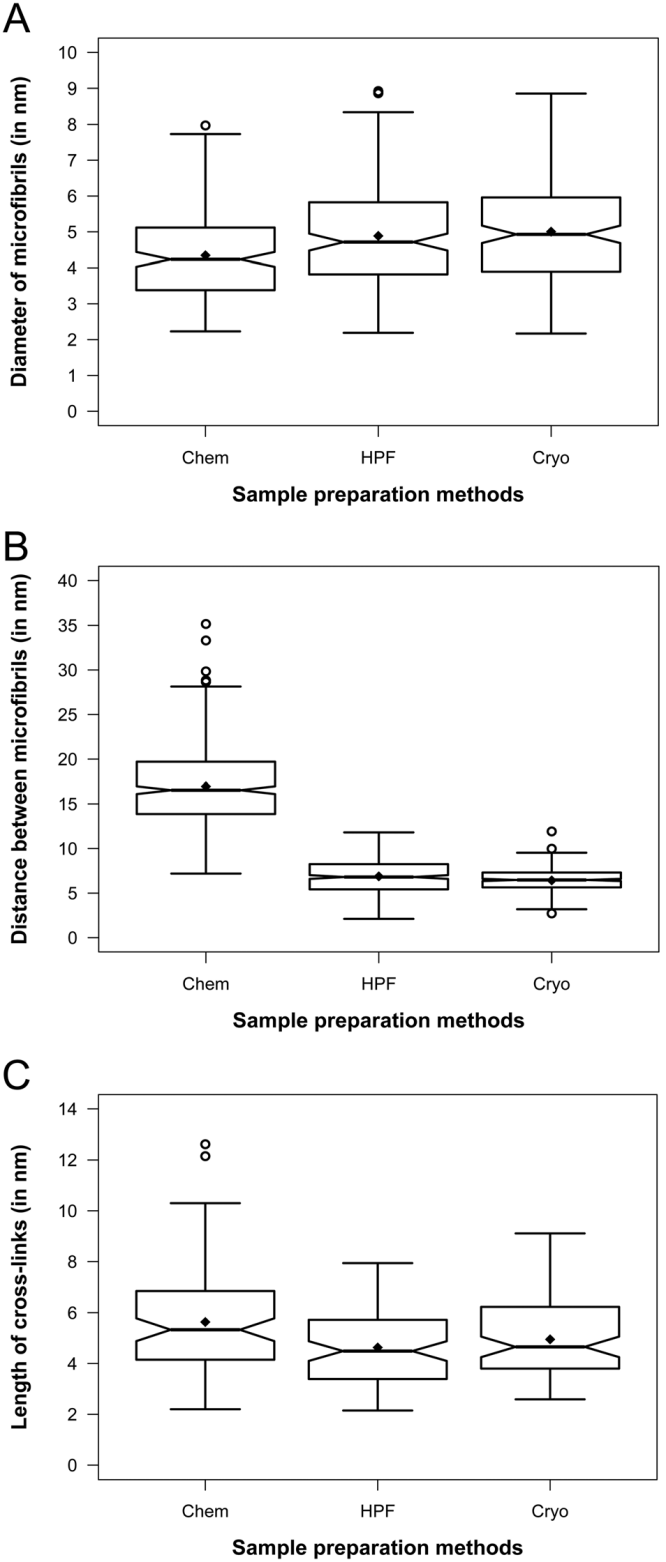
Notched boxplots showing quantitative comparison of cell wall preservation quality in electron tomograms. Cryo = Cryo-tomograms of vitreous sections. HPF = Tomograms of high pressure frozen, freeze-substituted samples. Chem = Tomograms of chemically prepared samples. The thick band inside the box is the median, and the bottom and top of the box are the first quantile (Q1) and the third quantile (Q3), respectively. The ends of the whiskers represent the range of data within 1.5 *IQR (Interquartile range; IQR = Q3−Q1) from the lower quantile (Q1) or the upper quantile (Q3). The notch displays deviation around the median ±1.57×IQR/sqrt of n (where n is the sample size), and approximately shows the 95% confidence interval of median, so that if the notches of two boxes do not overlap, their medians are usually significantly different. The diamond (⧫) represents the mean, and the circles (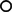) represent any outliers. A. Diameter of microfibrils, n = 180. B. Edge-to-edge distance between microfibrils, n = 450. C. Length of cross-links, n = 90.

We found that on average, the microfibrils in chemically prepared samples were slightly thinner than HPF-FS and cryo sample (Fig. 5A), but overall all three approaches match well within the accuracy limitations of electron tomographic imaging. The diameter measurements ranged between ∼2.2–8.0 nm (average 4.3±1.3 nm, n = 180) for chemically prepared samples, ∼2.2–8.9 nm (average 4.9±1.4 nm, n = 180) for the HPF-FS samples, and ∼2.2–8.9 nm (average 5.0±1.5 nm, n = 180) for the cryo samples. Hence, we conclude that filament diameter only differ slightly within the experimental error limit and thus agree well among all of the three sample preparation approaches. When examining the narrowest gap (edge-to-edge distance) between two neighboring microfibrils, we found that for conventionally prepared samples, the gap ranged between 7.2–35.2 nm (average 17.0±4.3 nm, n = 450), whereas in HPF-FS samples and cryo samples, such gaps were significantly narrower, i.e. 2.1–11.8 nm (average 6.9±2.1 nm, n = 450) and 2.7–11.9 nm (average 6.4±1.2 nm, n = 450) respectively (Fig. 5B). The corresponding center-to-center inter-microfibrilar distance ranged from 12.7–40.7 nm (average 22.5±4.3 nm, n = 450), 7.6–17.3 nm (average 12.4±2.1 nm, n = 450) and 8.2–17.4 nm (average 11.9±1.2 nm, n = 450), for chemically prepared, HPF-FS and cryo samples, respectively. The length of cross-links in chemically prepared samples ranged between 2.2–12.6 nm (average 5.6±2.1 nm, n = 90) in chemically prepared samples. Cross-links in HPF-FS and cryo samples were relatively shorter and comparable to each other ranging between 2.2–8.0 nm (average 4.6±1.5 nm, n = 90) in the HPF-FS samples and 2.6–9.1 nm (average 5.0±1.5 nm, n = 90) in cryo-sectioned samples (Fig. 5C). These quantitative data support our qualitative findings from visual inspection that suggested much better preservation of cell wall material in cryo-immobilized samples compared to conventionally processed samples, with remarkably close values for the HPF-FS and cryo-sectioned samples.

### Application of electron tomography in cell wall mutant characterization

**Figure 6:**
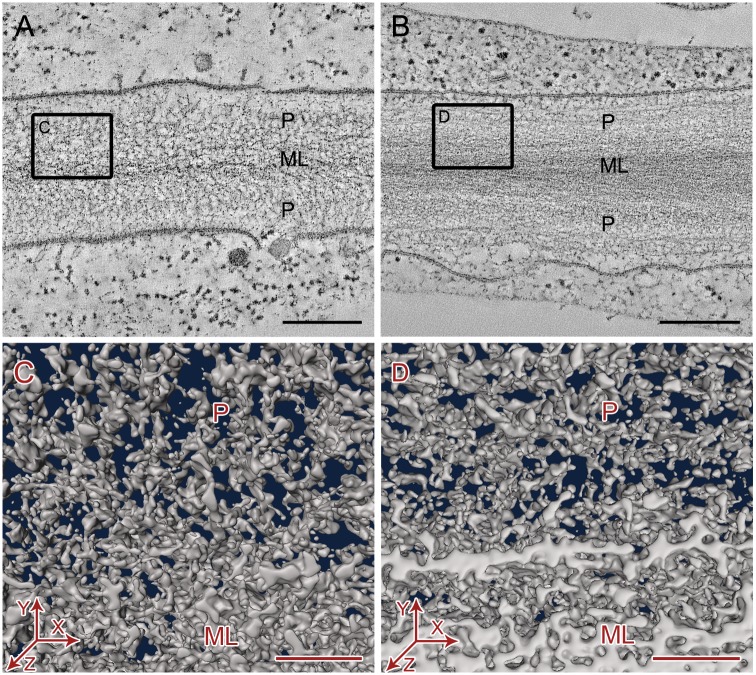
Qualitative comparison of primary cell wall ultrastructure in electron tomograms of high pressure frozen, freeze-substituted wild type hypocotyl parenchyma cells of wild type Arabidopsis (A, C) and cob-6 mutant (B, D). A–B. Slices of electron tomogram showing overview of cell wall ultrastructure in 2D. Bars = 250 nm. C–D. Small volumes of segmented cell wall tomograms showing organization of cell wall components from top view (XY plane). Bars = 50 nm. ML = Middle lamella, P = Primary cell wall.

**Figure 7:**
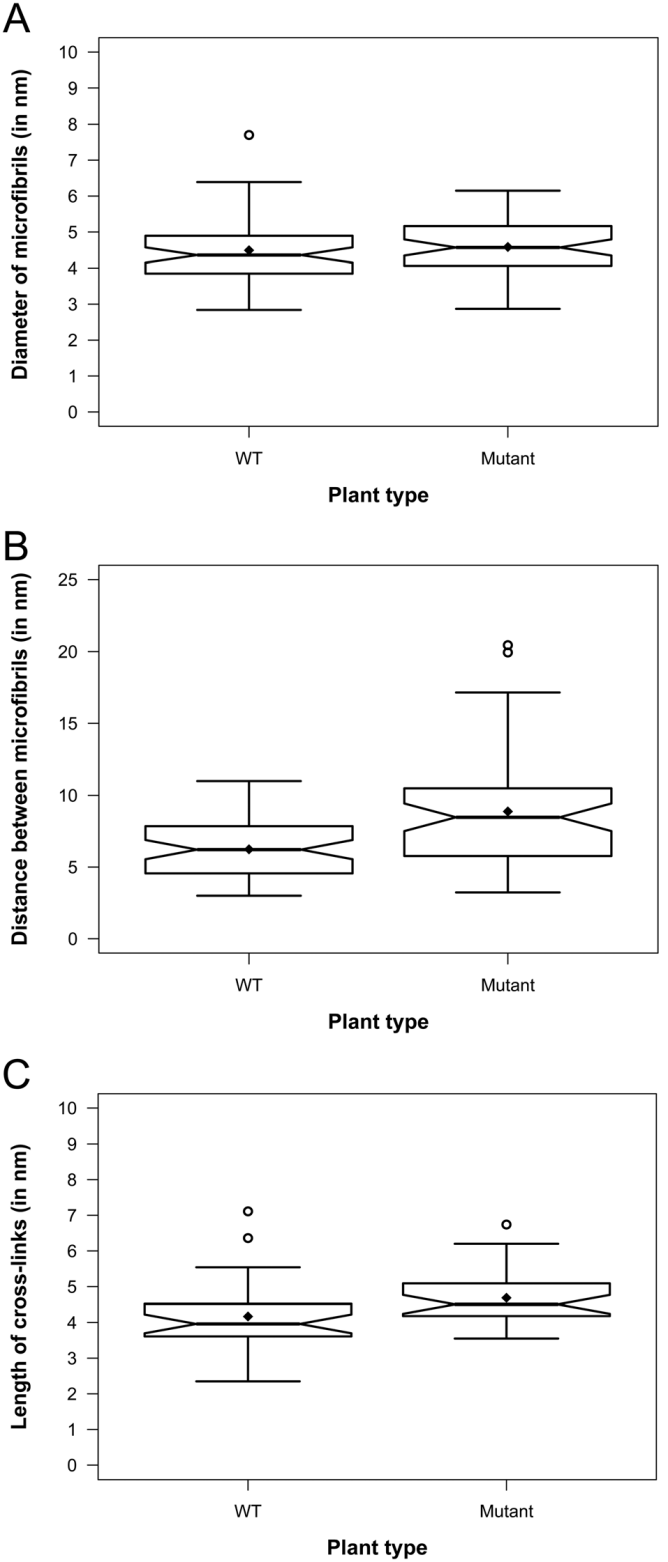
Notched boxplots showing quantitative comparison of primary cell wall ultrastructure in electron tomograms of high pressure frozen, freeze-substituted hypocotyl parenchyma cells of wild type Arabidopsis (WT) and *cob-6* (Mutant) plants. The thick band inside the box is the median, and the bottom and top of the box are the first quantile (Q1) and the third quantile (Q3), respectively. The ends of the whiskers represent the range of data within 1.5 *IQR (Interquartile range; IQR = Q3−Q1) from the lower quantile (Q1) or the upper quantile (Q3). The notch displays deviation around the median ±1.57×IQR/sqrt of n (where n is the sample size), and approximately shows the 95% confidence interval of median, so that if the notches of two boxes do not overlap, their medians are usually significantly different. The diamond (⧫) represents the mean, and the circles (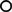) represent any outliers. A. Diameter of microfibrils, n = 60. B. Edge-to-edge distance between microfibrils, n = 60. C. Length of cross-links, n = 30.

Encouraged by our finding that HPF-FS approach provided comparable quality of preservation for primary cell wall compared to the technically challenging cryo-sectioning gold standard approach, we applied the HPF-FS method for structural characterization of a cell wall mutant, *cob-6*. Mutations in the *COBRA (COB)* gene of Arabidopsis have been reported to cause disorganization of cellulose microfibril orientation and reduction of crystalline cellulose in the cell walls of roots [54]–[56]. In our electron tomography study, we observed that in comparable hypocotyl parenchyma cells of wild type (WT) Arabidopsis and *cob-6* mutant, the cell walls, more noticeably the middle lamella, appeared less stained in the WT (Fig. 6A) compared to the mutant (Fig. 6B), indicating possible alteration in pectin composition. The cell membranes were loosened from the cell wall at several places in the mutant (Fig. 6B) suggesting possible disorganization at the wall-membrane interface. Visual inspections of the segmented tomograms (Fig. 6C–D) did not reveal any remarkable difference in the overall organization of cellulose microfibrils and hemicellulose cross-links in the primary cell walls of the mutant (Fig. 6D) compared to that of the WT (Fig. 6C). However, quantitative analysis of tomogram slices revealed that the microfibril diameters were comparable in both samples, ranging from ∼2.8–7.7 nm (average 4.5±0.9, n = 60) for the WT and ∼2.9–6.2 nm (average 4.6±0.8 nm, n = 60) for the mutant (Fig. 7A). However, the gaps between the microfibrils in the mutant primary walls were more random ranging between ∼3.2–20.6 nm (average 8.9±3.8 nm, n = 60) compared to a significantly narrower range of ∼3.0–11.0 nm (average 5.2±2.0 nm, n = 60) for the WT walls (Fig. 7B). Cross-links between microfibrils were slightly longer on average in the mutant wall, ranging between ∼3.6–6.8 nm (average 4.7±0.8 nm, n = 30) compared to the range of ∼2.4–7.1 nm (average 4.2±0.8 nm, n = 30) for the WT wall (Fig. 7C). Our results show that in comparison to the WT Arabidopsis, the diameter of cellulose microfibrils in primary cell walls essentially remain unchanged in the *cob-6* mutant, indicating no significant change in their individual macromolecular structures. However, the quantitatively detected increase in distances between the microfibrils and cross-link length indicate a subtle but noteworthy loss of microfibril organization in the mutants.

## Discussion

Conventional sample preparation protocols for RT electron tomography consist of chemical fixation, RT dehydration, RT resin embedding, heat polymerization, followed by RT ultrathin sectioning and RT electron tomography imaging, and this approach is the most widely used and the least technically challenging protocol currently available for studying molecular resolution 3D ultrastructure of biological samples. Variations of the conventional protocol have been used for all electron tomography studies on plant cell walls reported to date [49]–[52]. The conventional protocols do not require any expensive equipment for freezing, and tilt series data of samples prepared by this method can be easily acquired on most TEM microscopes that allow stage tilting. Conventional protocols are more commonly done on bench-top and are rather lengthy for plant samples due to slow infiltration of fixatives and resins through cell walls. Using microwave-assisted protocols significantly decreases the time required for each step with preservation being comparable to the bench-top protocols [81]. Using an automated microwave additionally makes the conventional protocol even less labor intensive, and allows a large number of samples to be prepared simultaneously. We consistently obtained fair preservation of Arabidopsis stem tissue samples with our 3-day protocol of microwave-assisted chemical fixation, dehydration and resin embedding. However, compared to cryogenically preserved samples the cell walls visually appeared to be more extracted in these samples, which was confirmed by the quantitative analysis. Such large-scale quantitative analyses of cell wall parameters were not performed in any of the previous electron tomography studies of plant cell wall, although a small-scale quantitative analysis [49] reported the diameter of crystalline cellulose core of a microfibril to be ∼2.2 nm and that with a non-crystalline outer layer to be ∼3.2 nm. The smallest diameter of microfibril we detected was also ∼2.2 nm and a large population of microfibrils we measured in our chemically prepared samples were ∼3 to 3.5 nm. We also detected microfibrils with a larger diameter that might include outer layers of non-cellulosic components such as hemicellulose and/or pectins, but overall the microfibrils in chemically prepared samples were slightly smaller in diameter compared to the HPF-FS and cryo samples. Although such differences are well within the resolution limit of the experimental technique and hence may or may not reflect true differences in composition, these differences in diameter greatly resemble the difference between microfibril from normal and pectin extracted cell walls of Arabidopsis imaged in an atomic force microscopy (AFM) study, where the microfibrils in pectin extracted cell wall were reported to be 3.2±0.13 nm while those from normal walls were reported to be 5.8±0.17 nm [44]. A more striking observation was the breakdown in consistency for microfibril orientations in many parts of the sample, as well as the drastically wider space between microfibrils with some of the inter-microfibrilar cross-connections being longer compared to those of the HPF-FS and cryo samples. The larger inter-microfibrillar distances in conventionally prepared samples suggest loosening and separation of the microfibril layers, possibly due to extraction of cell wall components that are usually loosely attached to the cellulose microfibrils as well as those that are present in the space between the cellulosic frameworks (such as pectins, hemicelluloses, and glycoproteins) during the lengthy dehydration and resin infiltration steps. The longer cross-links could possibly result from stretching during separation of the microfibril layers. The primary fixative, glutaraldehyde, as well as the secondary fixative/staining reagent, osmium tetroxide, usually target proteins and lipids in biological samples and hence are less effective for carbohydrates [53], [82]. The poorly fixed carbohydrates may thus leach out into the organic solvents during the lengthy dehydration steps. Conventional sample preparation protocols that include chemical fixation and dehydration are known to cause aggregation and extraction artifacts [53]. Our results suggest that such protocols are not reliable for studying the high-resolution ultrastructure of plant cell walls by electron tomography.

Cell wall structures were arguably preserved closest to their native state in samples prepared by the cryo method, as the tissue is not subjected to any fixatives, dehydration or resins, and thus is prepared only by self-pressurized rapid freezing (SPRF) of the stem tissue in capillary copper tubes with dextran. However, as the entire cryo-protocol including SPRF, vitreous sectioning of the frozen samples, and imaging by cryo-electron tomography needs to be carried out at liquid nitrogen temperature, the process is technically extremely challenging. Each individual step requires intensive training using specialized equipments, some of which are still in the stage of technology development. Cryo-tomography, while a mature field, is mostly done on whole-mount samples such as small cells (e.g. virus, bacteria) or isolated cell parts (organelles, protein complexes). However, this approach is almost never done on actual tissues, plant or animal, where orientation issues and operator skill development are often required. Though conventional high pressure freezing can solve these problems to some extent, the small sample in the extremely short high pressure freezer hats (100–200 µm deep) made cryo-sectioning very challenging. We found the SPRF in copper capillary tubes to be the most effective way to overcome all of the above mentioned issues, as this method allowed better tracking of tissue orientation and also provided enough sample to be sectioned and screened. By inserting the plant samples in the capillary tubes, quickly introducing 20% dextran solution into the tube, and then closing the tube from each of the ends we were able to remove excess air around the plant sample and create a uniform pressure buildup within the tubes upon rapid plunge-freezing into liquid ethane. Although our cryo-sections still suffered from non-flatness, occasional breaks, knife marks and ice contamination, good areas with intact cell walls could be imaged successfully. However, we could collect only single-axis tilt series from +60° to −60°, with 2° increments for the cryo-sections, as the vitreous sections of Arabidopsis tissue were very unstable even at the low dose used. The cell wall areas started boiling off if subjected to longer data collection times. Collecting dual-axis tilt series with 1° increment comparable to the resin tomograms was not possible as it would have required 1/4^th^ of the dose used, which would further decrease the contrast of the already low-contrast cryo-tomograms. Collecting dual-axis cryo tilt series would also require the region of interest to be away from the grid bars, which was logistically difficult with the mesh grids used for increasing stability of vitreous sections. Reconstruction of our single-axis cryo-tomograms was more difficult than for the dual-axis resin tomograms due to the low contrast, non-flatness and absence of gold fiducials in the tilt-series obtained by the cryo method. Reconstructing tomograms from a dual-axis series would require having flat sections with gold fiducials for accurate alignment combination of the two series. In spite of the low contrast of the tilt series images, segmentation, analysis and measurement of the various cell wall components was surprisingly straightforward in the cryo samples, possibly due to low background noise and lack of any additional electron dense material such as stains, thus providing sufficient contrast of each reconstruction slice. Some caution was needed to detect knife marks in the sample, in order to not confuse knife marks with inter-microfibrillar space, but fortunately, knife marks were found to occur in a direction clearly different from microfibril organization. The final segmented volumes revealed highly organized 3D architecture indicating that the fine structures of cell wall components were well preserved, as close as possible to their native state. The average microfibril diameter recorded in our cryo-sections was 5.0±1.5 nm, which matches well with the 5.8±0.17 nm measurement obtained by AFM of cell walls from Arabidopsis callus tissue [44]. While the cryo-sectioning approach is currently being instrumental to us in developing accurate and comprehensive cell wall models of low-lignin primary as well as lignin-rich secondary cell walls, we think it will be an unnecessarily complicated approach if the objective of a project is to characterize just primary or any lignin-less cell walls.

The HPF-FS method consistently produced high quality preservation of primary cell walls in the Arabidopsis stem tissue samples in our hands. Compared to the chemically prepared samples, the cell walls visually appeared denser, the overall organization of the microfibrils and layers within the cell wall appeared to be more consistent, the spaces between microfibrils were significantly narrower, and cross-connections between microfibrils were shorter and slightly thicker in the HPF-FS samples. Our analyses show that regarding all quantitative volumetric parameters as well as the overall level of order, HPF-FS data compare well with the cryo-sectioning data (typically considered as the gold standard for biological sample preservation), and yet is technically far less challenging than cryo-sectioning. Sectioning and imaging of HPF-FS samples at RT is much easier as resin sections allow cutting well-adherent sections, application of gold fiducial markers for improved alignment of the tilt series images, and dual axis data collection for reducing the data anisotropy. Furthermore, the ability to prescreen the samples for areas of interest as well as storage of the section for subsequent imaging, combined with what appears to be exquisite preservation render the HPF-FS method as an excellent choice for routine cell wall characterization. A major limitation of HPF-FS samples, however, is that unlike cryo-sections, the fine structures of middle lamella and secondary cell walls could not be clearly distinguished in these samples. Presence of high amounts of pectins in the middle lamella of young plants causes deep staining of the middle lamella due to higher affinity of ruthenium red stain for pectins [83]. The fine structure of middle lamella cannot be resolved well in HPF-FS due to the heavy metal staining, particularly if a threshold value is chosen for simultaneous visualization of cell wall components in the primary cell wall. On the other hand, the presence of significant amounts of lignin in secondary cell wall seems to make the walls appear featureless, possibly because lignin may serve as a penetration barrier for the stain and thus prevent contrast generation in the resin sections. Lignin is structurally similar to phenolic compounds present in epoxy resins [84], which could also be the reason of the apparent lack of texture and hence the perceived structural homogeneity of the resin-embedded lignin-rich secondary cell walls. The HPF-FS method can be used as a routine approach for studying cell walls with little or no lignin such as in parenchyma or collenchyma cells, lignin-less pteridophytes, bryophytes, algae, and also pretreated biomass samples. Most laboratories around the world that are familiar with TEM sample preparation can master the HPF-FS method much more easily compared to learning cryo-tomography of vitreous sections. As high-pressure freezing devices are becoming more readily available, and freeze-substitution can be carried out in a homemade low-cost setups [85]–[86], we submit that HPF-FS may be an appropriate compromise if the objective of a project is to characterize only primary cell walls or any lignin-less cell walls.

To this end, our comparison of WT Arabidopsis and *cob-6* cell wall mutant is a proof-of-concept and demonstrates our ability to detect relatively subtle changes in cell wall architecture. When all the technology and expertise were in place, the comparison of WT to mutant did not take longer than 3 weeks of experimental work, including sample preparation, imaging and image analysis. The whole process could be completed faster (within ∼1 week) if recently developed protocols of ‘quick freeze-substitution’, ‘rapid resin embedding and polymerization’ [85]–[87] are used. The *cob* mutants have been shown to display disorganization in cell wall microfibrils in their root cells by FESEM [55]. We detected and quantified a relatively subtle but significant microfibril disorganization in the parenchyma cells of hypocotyl samples by measuring the gaps between individual microfibrils. The powerful combination of localized qualitative and quantitative image analysis possible on the 3D tomography data allowed us to detect and quantify these subtle changes in the mutant wall organization, which cannot be detected by any other imaging method currently used for studying cell wall mutants.

## Conclusion

Electron tomography of cryo-immobilized plant tissue can reveal never-seen-before details of the 3D architecture of plant cell walls at macromolecular resolutions, which can be analyzed both qualitatively and quantitatively. Vitreous sectioning of fast-frozen samples provides closest-to-native preservation of both the lignin-less primary and lignin-rich secondary cell walls. Samples prepared by high-pressure freezing, freeze substitution and resin embedding (HPF-FS) also represented faithful preservation of the respective structures and the overall 3D organization of lignin-less primary cell walls. The HPF-FS approach is technically less demanding than the vitreous sectioning approach, yet is clearly superior to the commonly used bench-top or microwave assisted chemical protocols, which suffer from significant extraction artifacts. HPF-FS can hence, be widely adapted by the community as a routine tool for assessing cell walls with little or no lignin, such as in seedlings of cell wall mutants, lower plant groups and algae, and deconstructed biomass. Cryo-tomography of vitreous tissue sections should however be chosen if the goal of a project is to study the architecture of lignin-rich secondary cell walls and developing accurate and comprehensive 3D cell wall models.

## Supporting Information

Figure S1 **Comparison of segmentation approaches showing reliability of threshold segmentation.** A–D. Semi-automated threshold-based segmentation in Amira (A, C) compared with manual tracing of density in Amira (B, D). A–B. Slice of segmented tomogram in Amira. C–D. Segmented microfibrils in Amira. E–G. Microfibrils segmented by semi-automated threshold-based approach in Amira (E) compared with skeletons extracted from microfibrils segmented by algorithm-based automated approach (F, G). Bars (A–E, G) = 5 nm; (F) = 100 nm.
(TIF)

Figure S2
**Reliability of threshold segmentation approch for measument of microfibril diatemeter.** A. Insignificant difference in measurements with small variability in threshold value (n = 75). B. Shift of ∼1 nm only with a larger variability in threshold value (n = 75). C. Comparable results obtained by two independent users from segmenting and analyzing the same 3D volume (n = 60). D. Comparable results obtained from segmenting and analyzing three different tomograms of three different cell wall samples prepared by the same sample preparation method (n = 60). The thick band inside each notched box is the median, and the bottom and top of the box are the first quantile (Q1) and the third quantile (Q3), respectively. The ends of the whiskers represent the range of data within 1.5 *IQR (Interquartile range; IQR = Q3−Q1) from the lower quantile (Q1) or the upper quantile (Q3). The notch displays deviation around the median ±1.57×IQR/sqrt of n (where n is the sample size), and approximately shows the 95% confidence interval of median, so that if the notches of two boxes do not overlap, their medians are usually significantly different. The diamond (⧫) represents the mean, and the circles (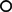) represent any outliers.(TIF)

Figure S3 **Chemically extracted Arabidopsis cell walls to remove hemicelluloses and pectins.** A. Slice of electron tomogram showing overview of extracted cell wall. Bar = 250 nm. B. Sub-area of tomogram. Bar = 100 nm. C. Segmented cell wall showing orientation of filamentous cellulose microfibrils. No cross-connections detected. Bar = 10 nm.
(TIF)

Video S1 **The process of cryo-sectioning of frozen samples and collection of vitreous sections on a EM grid.**
(MOV)

Video S2 **A reconstructed 3D volume (tomogram) of an Arabidopsis cell wall showing a z-stack of ∼1** **nm thin slices.**
(MOV)

Video S3 **The process of threshold segmentation in Amira, to obtain a segment map that include most of the electron-dense features above background noise for each slice, and rendering as a mesh surface for 3D visualization and further analyses.**
(MOV)
