# Supplementary figures and images for: Electron Tomography of Cryo-Immobilized Plant Tissue: A Novel Approach to Studying 3D Macromolecular Architecture of Mature Plant Cell Walls In Situ
Source: PLoS One. 2014 Sep 10;9(9):e106928. doi: 10.1371/journal.pone.0106928 (PMC4160213; doi:10.1371/journal.pone.0106928)

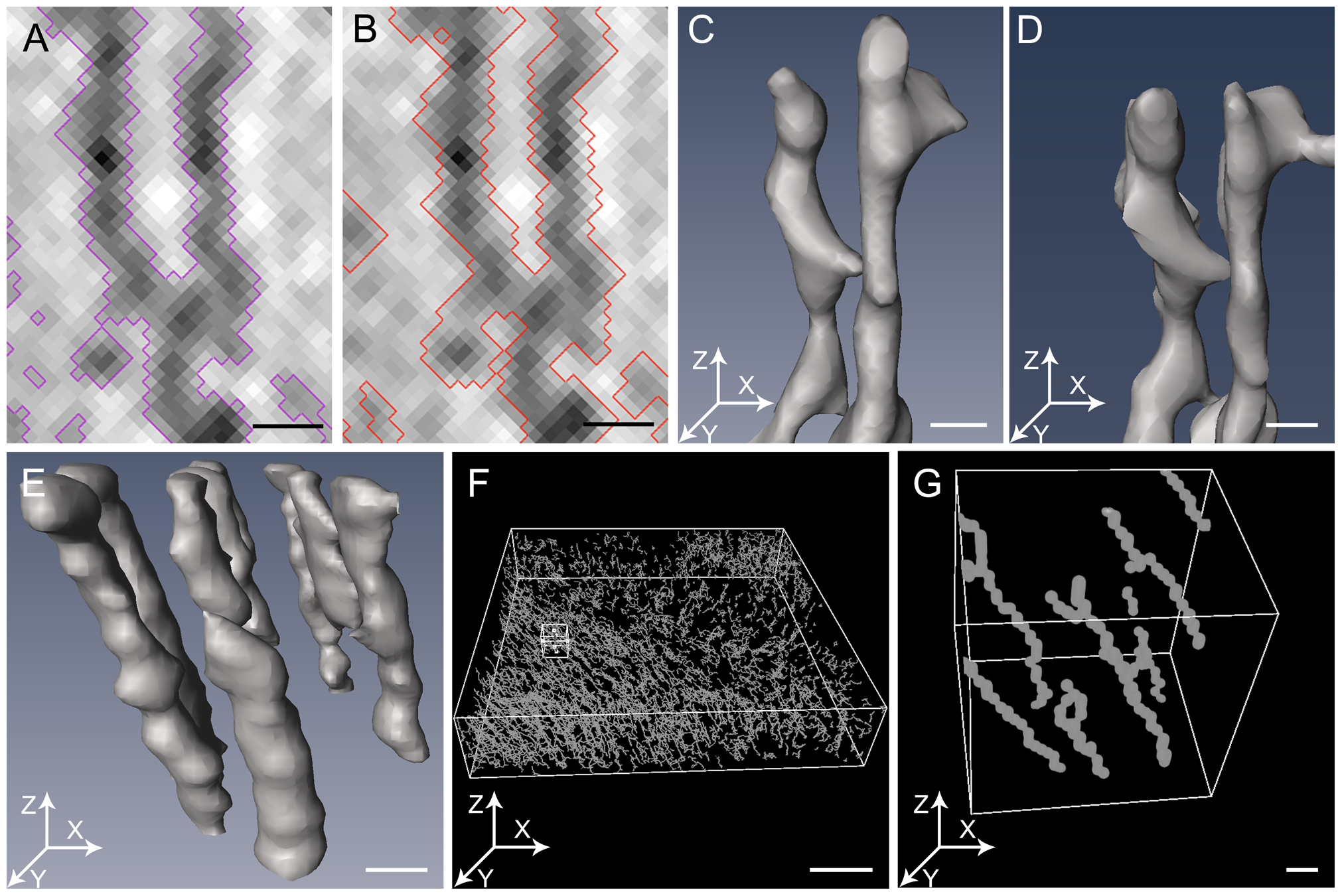

Supplement: Figure S1 — Comparison of segmentation approaches showing reliability of threshold segmentation. A–D. Semi-automated threshold-based segmentation in Amira (A, C) compared with manual tracing of density in Amira (B, D). A–B. Slice of segmented tomogram in Amira. C–D. Segmented microfibrils in Amira. E–G. Microfibrils segmented by semi-automated threshold-based approach in Amira (E) compared with skeletons extracted from microfibrils segmented by algorithm-based automated approach (F, G). Bars (A–E, G) = 5 nm; (F) = 100 nm. (TIF) [file pone.0106928.s001.tif]

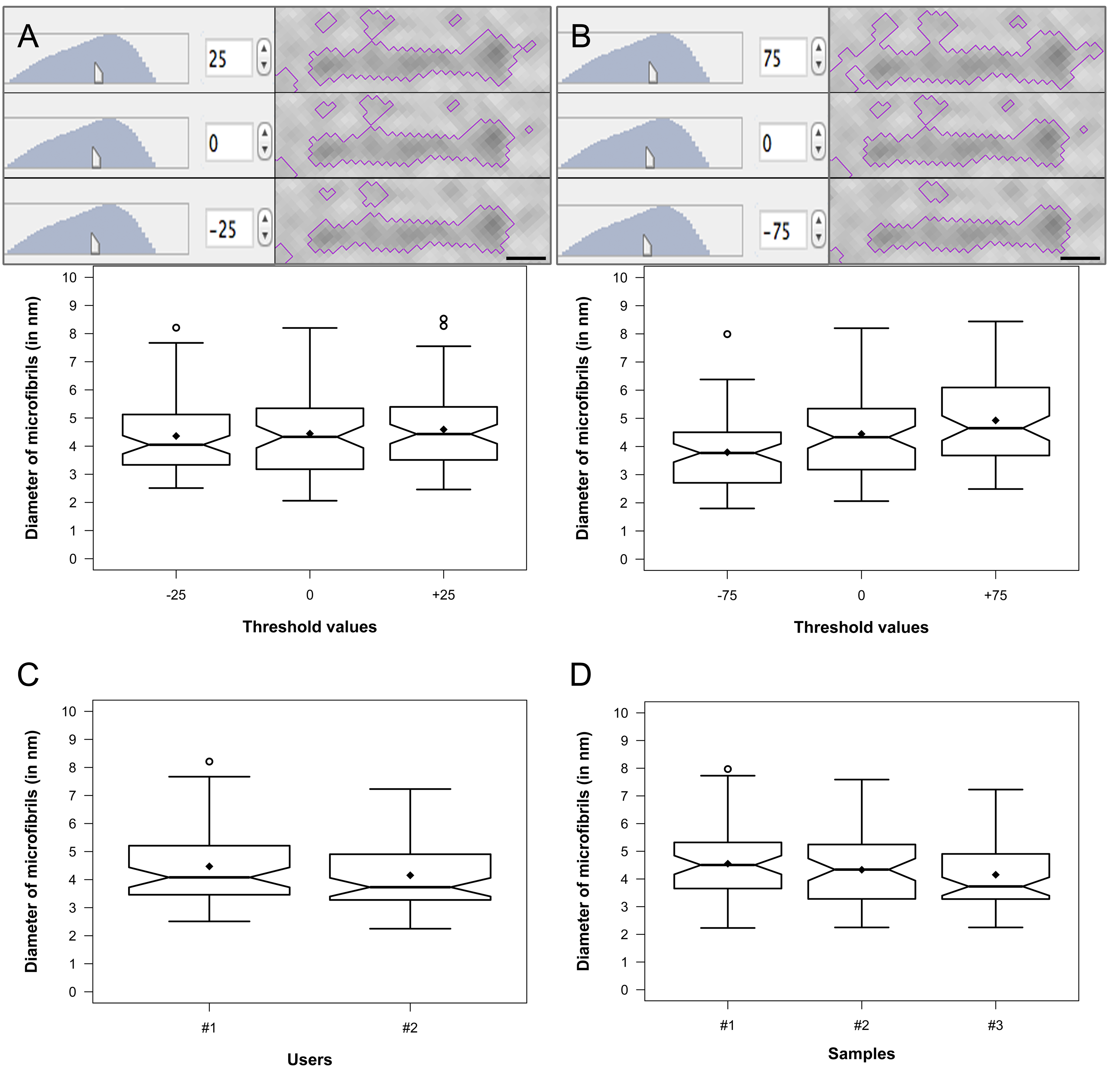

Supplement: Figure S2 — Reliability of threshold segmentation approch for measument of microfibril diatemeter. A. Insignificant difference in measurements with small variability in threshold value (n = 75). B. Shift of ∼1 nm only with a larger variability in threshold value (n = 75). C. Comparable results obtained by two independent users from segmenting and analyzing the same 3D volume (n = 60). D. Comparable results obtained from segmenting and analyzing three different tomograms of three different cell wall samples prepared by the same sample preparation method (n = 60). The thick band inside each notched box is the median, and the bottom and top of the box are the first quantile (Q1) and the third quantile (Q3), respectively. The ends of the whiskers represent the range of data within 1.5 *IQR (Interquartile range; IQR = Q3−Q1) from the lower quantile (Q1) or the upper quantile (Q3). The notch displays deviation around the median ±1.57×IQR/sqrt of n (where n is the sample size), and approximately shows the 95% confidence interval of median, so that if the notches of two boxes do not overlap, their medians are usually significantly different. The diamond (⧫) represents the mean, and the circles () represent any outliers. (TIF) [file pone.0106928.s002.tif]

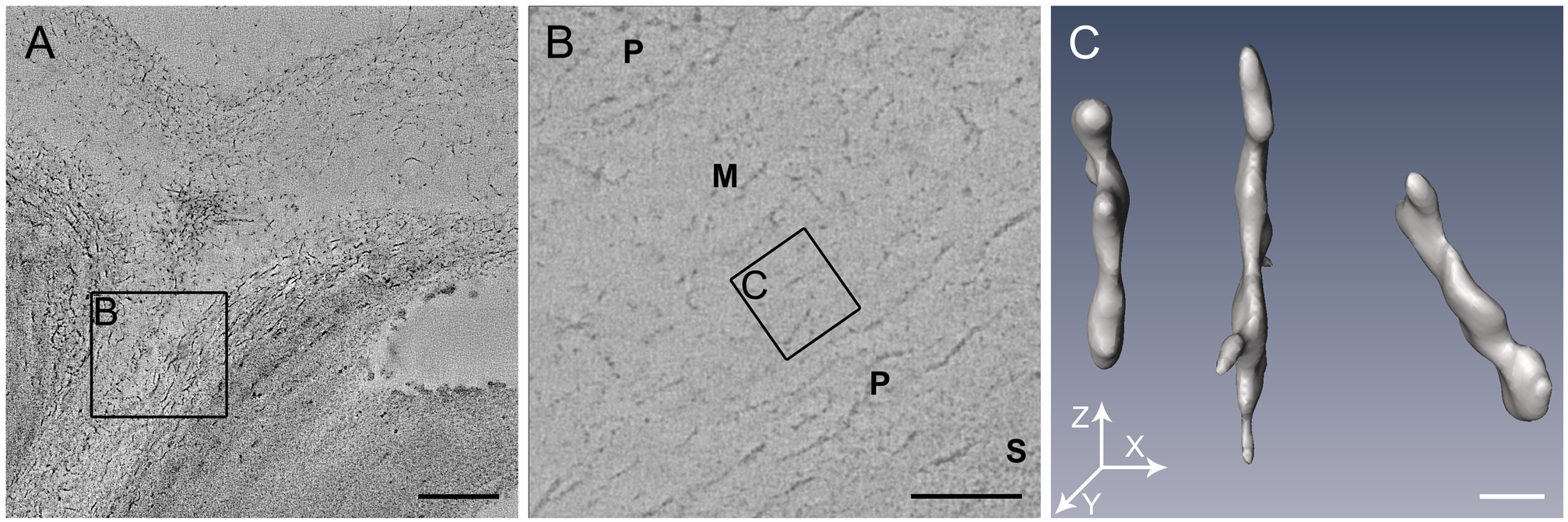

Supplement: Figure S3 — Chemically extracted Arabidopsis cell walls to remove hemicelluloses and pectins. A. Slice of electron tomogram showing overview of extracted cell wall. Bar = 250 nm. B. Sub-area of tomogram. Bar = 100 nm. C. Segmented cell wall showing orientation of filamentous cellulose microfibrils. No cross-connections detected. Bar = 10 nm. (TIF) [file pone.0106928.s003.tif]
